# Health Risk Assessment for Air Pollutants: Alterations in Lung and Cardiac Gene Expression in Mice Exposed to Milano Winter Fine Particulate Matter (PM2.5)

**DOI:** 10.1371/journal.pone.0109685

**Published:** 2014-10-08

**Authors:** Giulio Sancini, Francesca Farina, Cristina Battaglia, Ingrid Cifola, Eleonora Mangano, Paride Mantecca, Marina Camatini, Paola Palestini

**Affiliations:** 1 Department of Health Science, POLARIS Research Center, University of Milano-Bicocca, Monza, Italy; 2 Department of Medical Biotechnologies and Translational Medicine (BIOMETRA), Università degli Studi di Milano, Segrate, Italy; 3 Department of Environmental Science, POLARIS Research Center, University of Milano-Bicocca, Milano, Italy; 4 Institute of Biomedical Technology, CNR, Segrate, Italy; Utah State University, United States of America

## Abstract

Oxidative stress, pulmonary and systemic inflammation, endothelial cell dysfunction, atherosclerosis and cardiac autonomic dysfunction have been linked to urban particulate matter exposure. The chemical composition of airborne pollutants in Milano is similar to those of other European cities though with a higher PM2.5 fraction. Milano winter fine particles (PM2.5win) are characterized by the presence of nitrate, organic carbon fraction, with high amount of polycyclic aromatic hydrocarbons and elements such as Pb, Al, Zn, V, Fe, Cr and others, with a negligible endotoxin presence. In BALB/c mice, we examined, at biochemical and transcriptomic levels, the adverse effects of repeated Milano PM2.5win exposure in lung and heart. We found that ET-1, Hsp70, Cyp1A1, Cyp1B1 and Hsp-70, HO-1, MPO respectively increased within lung and heart of PM2.5win-treated mice. The PM2.5win exposure had a strong impact on global gene expression of heart tissue (181 up-regulated and 178 down-regulated genes) but a lesser impact on lung tissue (14 up-regulated genes and 43 down-regulated genes). Focusing on modulated genes, in lung we found two- to three-fold changes of those genes related to polycyclic aromatic hydrocarbons exposure and calcium signalling. Within heart the most striking aspect is the twofold to threefold increase in collagen and laminin related genes as well as in genes involved in calcium signaling. The current study extends our previous findings, showing that repeated instillations of PM2.5win trigger systemic adverse effects. PM2.5win thus likely poses an acute threat primarily to susceptible people, such as the elderly and those with unrecognized coronary artery or structural heart disease. The study of genomic responses will improve understanding of disease mechanisms and enable future clinical testing of interventions against the toxic effects of air pollutant.

## Introduction

The first evidence of a link between short term exposure to air pollution and increased mortality dates to the Meuse Valley in Belgium of 1930 and to the London “great smog” of 1952 [1], [2], but in the last years a growing number of studies correlated high levels of acute air pollution exposure to increased rate of hospital admission for cardiovascular events. Short term exposures to PM10 (particles ≤10 µm in aerodinamic diameter) and to PM2.5 (particles ≤2.5 µm in aerodinamic diameter) have been connected to higher hospitalization risk for congestive heart failure, myocardial infarction and acute coronary syndrome [3]. Moreover, large scale long term studies demonstrated a close relationship between PM2.5 exposure, lung cancer and cardiopulmonary mortality [4], [5], [6].

Pathways leading to cardiovascular effects of particulate matter exposure have been mainly linked to oxidative stress, pulmonary and systemic inflammation, endothelial cell dysfunction, atherosclerosis and altered cardiac autonomic function [7]. PM2.5 fraction toxicity was emphasized because of particles deposition into the deep airways and terminal alveoli, chemical composition, indoor penetration and prolonged atmospheric lifetime [8].

Various kind of chemicals are adsorbed onto fine particulate matter collected during winter season, such as trace of metals and polycyclic aromatic hydrocarbons (PAHs) [9], [10], [11], [12]. These chemicals are known to dissolve and translocate into blood circulation after particles deposition in the lungs. Some of these metals initiate redox reactions producing reactive oxygen species, implicated in inflammation and adverse health effects [13], thus the specific chemical composition seems to be the most important issue to determine adverse health effects [8]. Many studies investigated the biological response after exposure to air pollutants at molecular, cellular and whole organism levels. It has been clearly established that air pollution, derived from a variety of sourcesis able to induce specific biological responses [14].

Moreover, genomic alterations play an important role in mediating pathogenic mechanisms sustained by air pollutants.

Mice are useful *in-vivo* models to study particulate matter induced toxicity. In a murine model of asthma by day 4 of exposure to particulate matter, microarrays detected 436 differentially expressed genes, with activated pathways concerning innate immunity, allergic inflammation, chemotaxis, complement system, inflammation, host defence and signal transduction, thus implicating air pollutant exposure to susceptibility and severity of asthma [15]. Furthermore, several studies evaluating gene expression in human cells lines (BEAS-2B and A549) showed up-regulation of inflammatory cytokines and mediator genes, STAT3 activation pathway and oxidative stress in response to PM2.5 or DEP (Diesel Exhaust Particles) exposure [16], [17], [18], [19].

Annual PM2.5 levels in Milano are greater than those observed in urban sites in Europe, while its chemical composition is similar to those of other European cities. Indeed Milano PM2.5win is mainly constituted by particles with a mean dimension ranging from 40 nm to 300 nm, and only a small number of particles exceeded 1 µm [10]. In particular, winter fine particles (PM2.5win) are characterized by the presence of nitrate, organic carbon fraction (with high amount of PAHs) and various elements (Pb, Al, Zn, V, Fe, Cr and others), while a negligible endotoxin presence has been found [10].

It has been suggested that PM2.5 may contribute to respiratory and cardiovascular morbidity and mortality, however, the molecular mechanism is still unknown. Here we focused on heart and lungs to examine the adverse effects induced by PM2.5win exposure in mice. Within lung and heart we analysed biomarkers associated to particulate matter exposure such as ET-1, Hsp70, Cyp1A1, Cyp1B1, OGG1, HO-1, MPO, Caspase3-p17, Caspase8-p18, p-H3 and H3. At gene expression levels we carried out a global gene expression profiling by GeneChip technology in heart and lungs. To discuss the biological meaning of gene expression changes induced by PM2.5win exposure we apply functional enrichments approaches by means of gene ontology and pathways analyses.

## Materials and Methods

### Animals

Male BALB/c mice (7–8 weeks old) were purchased from Harlan; food and water were administered ad libitum. Mice were housed in plastic cages under controlled environmental conditions (temperature 19–21°C, humidity 40–70%, lights on 7 a.m.–7 p.m.). Animal use and care procedures were approved by the Institutional Animal Care and Use Committee of the University of Milano-Bicocca and complied with guidelines set by Italian Ministry of Health (DL 116/92); invasive procedures have been performed under anaesthesia and all efforts were made to minimize suffering.

### PM sources and characterization

Atmospheric PM2.5win was collected during winter 2008 in Torre Sarca, an urban site in Milano, as previously described [10]. PM2.5win was sampled and chemical analyses were performed as described in Perrone et al. [12], [20]; Milano PM2.5win chemical composition (inorganic ions, elements and PAHs) is summarized in Tab.1.

**Table 1:** PM2.5win chemical composition.

INORGANIC IONS (µg/µg PM)		ELEMENTS (µg/µg PM)		PAHs (µg/µg PM)	
	mean		mean		mean
F^-^	0.00027	Al	0.00051	BaA	0.000014
Cl^-^	0.0134	As	0.00001	Cr	0.00002
NO_3_^-^	0.2880	Ba	0.00005	BeP	0.000034
PO_4_^3-^	0.0006	Cd	0.000007	Bb+jF	0.000056
SO_4_^2-^	0.0671	Cr	0.00003	BkF	0.000013
Na^+^	0.0022	Cu	0.00019	BaP	0.000023
NH_4_^+^	0.1277	Fe	0.00457	dBahA	0.000001
K^+^	0.0071	Mn	0.00007	BghiP	0.000015
Mg^2+^	0.0002	Mo	0.00003	IcdP	0.000022
Ca^2+^	0.0024	Ni	0.00003		
		Pb	0.00018		
		V	0.000017		
		Zn	0.00075		

Table summarizing mean chemical composition (µg/µg PM) of 4 PM2.5win pooled samples (modified by Perrone et al. [12]). Inorganic ions explained about the 50% of the PM mass, the sum of all elements explained about the 0.6% while the contribution of PAHs was 0.019%. BaA: benzo[a]anthracene; BeP: benzo[e]pyrene; Bb+jF: benzo[b+j]fluoranthene; BkF: benzo(k)fluoranthene; BaP: benzo[a]pyrene; dBahA: dibenzo[a,h]anthracene; BghiP: benzo[g,h,i]perylene; IcdP: indeno[1,2-Cd]pyrene.

Concerning sources, traffic and heating during cold season constitute the 49–53% of the primary combustion sources of fine PM; during warm season they constitute about the 25%, while secondary sources are predominant (50–66%) [20]. Elemental carbon (primarily from traffic) contributes for about 10–15% to the fine fraction; organic matter, calculated applying a specific organic matter-to-organic carbon conversion factor to each source, contributes for 31–38% to the fine fraction [20].

Particles were recovered from filters by sequential sonications (four cycles of 20 min each) in sterile water; detached particles were dried into a desiccator and weighed. Particles' suspensions were prepared as follow: just before the intratracheal instillation, PM2.5win aliquots were properly diluted in sterile saline, sonicated and vortexed and then immediately instilled in mice.

### Dose

The aim of this study is to disclose short-term adverse effects on respiratory and cardiovascular systems induced by winter fine particles exposure. Similar investigations have been previously based on very high PM exposure rate [11], [21], [22]. Starting from the dose proposed for repeated instillation protocol by Happo et al. [11] we reduced the cumulative dose of fine particulate matter to 0.3 mg/mouse within the same time points, in order to apply the same protocol proposed by Farina et al. [23]. The treatment scheme here proposed has been specifically outlined to rise extra-pulmonary adverse effects being lungs still affected.

### Intratracheal PM2.5win instillation

Animal testing was replicated twice by instilling intratracheally a total of 5 sham and 5 PM2.5win-treated mice. For gene expression profiling and histological analyses, we considered 5 sham and 5 PM2.5win-treated mice.

Male BALB/c mice were briefly exposed to 2.5% isoflurane (Flurane) and kept under anaesthesia during the whole instillation procedure. Once a deep stage of anaesthesia was reached, mice were intratracheally instilled by means of MicroSprayer Aerosolizer system (MicroSprayer Aerosolizer- Model IA-1C and FMJ-250 High Pressure Syringe, Penn Century, USA) with 100 µg of PM2.5win in 100 µl of isotonic saline solution, or 100 µl of isotonic saline solution (sham) as described in Mantecca et al. [24], [25] and in Farina et al. [26].

The intratracheal instillation was performed on days 0, 3 and 6, for a total of three instillations.24 h after the last instillation, mice from each experimental group were euthanized with an anesthetic mixture overdose (Tiletamine/Zolazepam-Xylazine and isoflurane). The broncho alveolar lavage (BAL) procedure, pellets and supernatant recovery have been performed as described in Mantecca et al. [24], [25].

### Bronchoalveolar lavage fluid analyses (BALf)

#### Cell counts

After centrifugation, total and differential cells counts were performed according to Mantecca et al. [24], [25] and Farina et al. [26].

#### Cytokines analyses

The analyses of pro-inflammatory cytokines and chemokines released within the BALf was performed by DuoSet ELISA kits for TNF-α, MIP-2 and IL-1β(R&D Systems, Minneapolis, MN) according to manufacturer's protocols.

#### Biochemical analyses

The following biochemical analyses were performed on cell-free BALf supernatants. The commercially available kits for ALP (DALP-250 QuantiChrom Alkaline Phosphatase Assay Kit, Gentaur Molecular) and LDH (DLDH-100 QuantiChrom Lactate Dehydrogenase Kit, Gentaur Molecular) were employed according to manufacturer's instructions.

#### Other proteins

A total of 30 µg of BALf proteins obtained from sham and PM2.5win-treated mice were loaded onto SDS-PAGE, submitted to electrophoresis followed by Western blot, and tested for MPO and Hsp70 (anti-MPO sc-16128 1∶200, anti-Hsp70 sc-1060 1∶200, Santa Cruz), according to the procedures described below.

### Lung and heart parenchyma protein markers analyses

For the detection and quantification of proteins, organs were minced at 4°C, suspended in NaCl 0.9%, briefly homogenized for 30 seconds at 11000 rpm with Ultra-Turrax T25 basic (IKA WERKE), then sonicated for other 30 seconds. Then samples were submitted to trichloroacetic acid (TCA) precipitation according to the procedure described by Farina et al. [26]. The pellets were suspended in water and protein amount measured by BCA method (Sigma Aldrich, USA).

Thereafter, lung and heart homogenates of sham and PM2.5win-treated mice were loaded onto SDS-PAGE and submitted to electrophoresis, followed by Western blot, according to procedures previously described [26]. Lung parenchyma was assessed with specific antibodies for ET-1 (sc-21625), Hsp70 (sc-1060), Cyp1A1 (sc-9828), Cyp1B1 (sc-32882), OGG1 (sc-12075), HO-1 (sc-10789), MPO (sc-16128-R), Caspase3-p17 (sc-22139), Caspase8-p18 (sc-7890), p-H3 (sc-8656-R) and H3 (sc-8654) (all 1∶200, Santa Cruz). Heart homogenates were incubated with specific antibodies for the same proteins evaluated in lungs. Then, blots were incubated for 1.5 h with horseradish peroxidase-conjugated anti-rabbit IgG (1∶5000, Pierce) or anti-goat IgG (1∶2000, Santa Cruz) diluted in PBS-Tween20/milk or in TBS-Tween20/BSA. Proteins were detected by ECL using the SuperSignal detection kit (Pierce, Rockford, IL). Immunoblot bands were analysed and the optical density (OD) quantified by KODAK (Kodak Image Station 2000R); all the data have been normalized to β-actin (1∶1500, Sigma) and each protein in PM2.5win-treated group has been normalized to respective sham group.

All these proteins have been screened in the lung parenchyma of mice submitted to gene expression and histology, in order to confirm PM2.5win exposure.

### Statistical analyses

Results have been expressed as mean ± standard error of the mean (s.e.). Data distribution was tested by Shapiro-Wilk test; statistical differences were tested accordingly by t-test or non-parametric U Mann-Whitney test. Statistical differences were considered to be significant at the 95% level (*p* value <0.05).

### Lung histological analyses

Lungs from sham and PM2.5win-treated mice were properly inflated, excised and immediately formalin fixed and processed for routine histology. Briefly, after being preserved for 24 h in the fixative, tissue samples were rinsed in distilled water, dehydrated in an ethanol series from 70% to 100% and embedded in Bio-plast tissue embedding medium. For each sham and PM2.5win exposed lung sample, 7 µm serial sections were cut by a rotary microtome, mounted on slides and stained with Mayer's haemalaun and alcoholic eosin. Samples were qualitatively screened by means of Zeiss Axioplan microscope at a magnification of 40× and images were taken using Zeiss AxioCam MRc5 digital camera interfaced with the Axiovision Real 4.6 software. Figure panels were prepared by means of Adobe Photoshop.

### Gene expression profiling by Affymetrix GeneChip

For RNA analyses, a total of 5 sham and 5 PM2.5win-treated mice were considered. Lungs, not submitted to BAL procedure (called “no-BAL”), have been excised, suspended in an appropriate volume of RNA Later and submitted to total RNA extraction. Total RNA was extracted from tissues (lung and heart) by means of miRNeasy extraction kit (Qiagen, Hilden, Germany), according to manufacturer's instructions. RNA samples were quantified by ND-1000 spectrophotometer (NanoDrop Technologies, Wilmington, DE, USA). RNA quality was checked by microcapillary electrophoresis with 2100 BioAnalyzer (Agilent Technologies, Santa Clara, CA, USA). Total RNA integrity was assessed on the basis of the RIN (RNA Integrity Number) factor. RNA samples were stored at −80°C until use. To perform a differential gene expression analyses comparing PM2.5win-treated mice to sham, we assessed gene expression levels in lung and hearth tissues by means of Affymetrix GeneChip technology. RNA samples were prepared and hybridized onto the GeneChip Mouse Gene 1.0 ST Array (Affymetrix, Santa Clara, CA, USA), which measures gene expression levels of 28,000 coding transcripts and 7,000 non-coding (include ∼2,000) long intergenic non-coding transcripts, by means of a single probe set per gene comprised of multiple probes distributed along the entire length of the genomic locus, thus offering a whole-transcript coverage. Mouse gene 1.0 ST Array probe design is based on the March 2006 human genome sequence assembly (UCSC hg18, NCBI Build 36). Starting from 100 ng of total RNA per sample, labelled targets were prepared using Ambion Whole Transcript (WT) Expression Kit (Applied Biosystems, Life Technologies) and GeneChip WT Terminal Labeling and Controls Kit (Affymetrix), following manufacturers' instructions. Briefly, 100 ng of total RNA was primed with synthetic primers containing a T7 promoter sequence and reverse transcribed into first-strand cDNA. Afterwards, the single-stranded cDNA is converted into double-stranded cDNA, using DNA Polymerase and RNase H to simultaneously synthesize second-strand cDNA and degrade the original RNA. The *in-vitro* transcription (IVT) reaction is then performed to synthesize and amplify the antisense cRNA. Next, the cRNA is purified and measured for yield and size distribution. 10 µg of cRNA are reverse transcribed using random primers, to synthesize second-cycle cDNA. The cRNA template is degradated by RNase H to leave a single-stranded cDNA, that is purified and assessed for size distribution. Lastly, 5.5 µg of cDNA is fragmented, biotin terminally labeled and hybridized for 16 hours at 45°C onto Gene 1.0 ST Array. The array is then washed and stained using the Affymetrix Fluidics Station FS-450. Fluorescent images of each array are acquired using Affymetrix GeneChip Scanner 3000 7 G and analyzed using GeneChip Operating Software (GCOS). Array data quality control was conducted using Affymetrix Expression Console (V 1.2). the data have been normalized by robust multiarray average (RMA) and log-2 transformed. The entire data set (20 samples, including four groups) were analysed by analyses of variance (ANOVA) using Partek GS (Partek Genomic Suite, St Louis, MO). To identify two lists of differentially expressed genes (DEGs) we compared the group of five PM2.5win-treated mice to five sham mice either for lung or for heart tissues on the basis of a cut off a 2 fold-change (FC) and a significance level of *p* value <0.01. Average linkage hierarchical clustering of DEG (row) and samples (column) has been performed by dChip software [27]. Data (CEL files) discussed in this publication have been deposited in ArrayExpress repository (http://www.ebi.ac.uk/arrayexpress/) and are available through the accession number E-MTAB-2751.

### Identifications of gene ontology categories and genes enrichment analyses

The identification of biological roles of DEGs were addressed using various tools and database such as the Database for Annotation, Visualization and Integrated Discovery (DAVID v6.) [28], GeneTrail database [29] and NanoMiner database [http://nanominer.cs.tut.fi/users/login]. We classifiedDEGs into Gene Ontology (GO) categories and pathways. In particular for each DEG list we focused the enrichment analyses on GO of molecular function (MF) terms and KEGG pathway. The categories with a *p* value <0.05 were considered significantly enriched.

## Results

### BALf analyses

All the biomarkers tested within the BALf of PM2.5win-treated mice disclosed no differences comparing to sham mice (Tab.2, A and B).

**Table 2:** BALf analyses.

		Sham		PM2.5win	
		mean	± s.e.	mean	± s.e.
**A**	Total cells (E+06)	2.9	0.99	3.5	0.71
	AMs%	80.87	3.85	73.72	9.39
	PMNs%	18.70	3.67	25.25	9.79
	Ls%	0.47	0.23	1.02	0.50
	TNF-α (pg/mL)	165.05	50.70	193.14	25.51
	MIP-2 (pg/mL)	172.97	40.23	217.36	32.32
	IL-1β (pg/mL)	65.59	6.68	101.92	20.50
	LDH (IU/L)	40.80	1.01	44.54	2.09
**B**	ALP (IU/L)	0.47	0.18	0.42	0.06
	MPO	1.00	0.28	1.47	0.24
	Hsp70	1.00	0.22	0.78	0.07
					

(A): table summarizing results of cell counts and biochemical analyses in BALf from sham and PM2.5win-treated mice, 24 h after the third intratracheal instillation. Statistical differences were tested accordingly by non-parametric U Mann-Whitney test. All the examined markers resulted unchanged comparing to sham.

(B): immunoblotting results in BALf from sham and PM2.5win -treated mice, 24 h after the third intratracheal instillation; each protein in PM2.5win-treated group has been normalized onto respective sham group. Statistical differences were tested accordingly by non-parametric U Mann-Whitney test. All the examined markers resulted unchanged comparing to sham.

### Lungs and heart parenchyma proteins analyses

**Figure 1:**
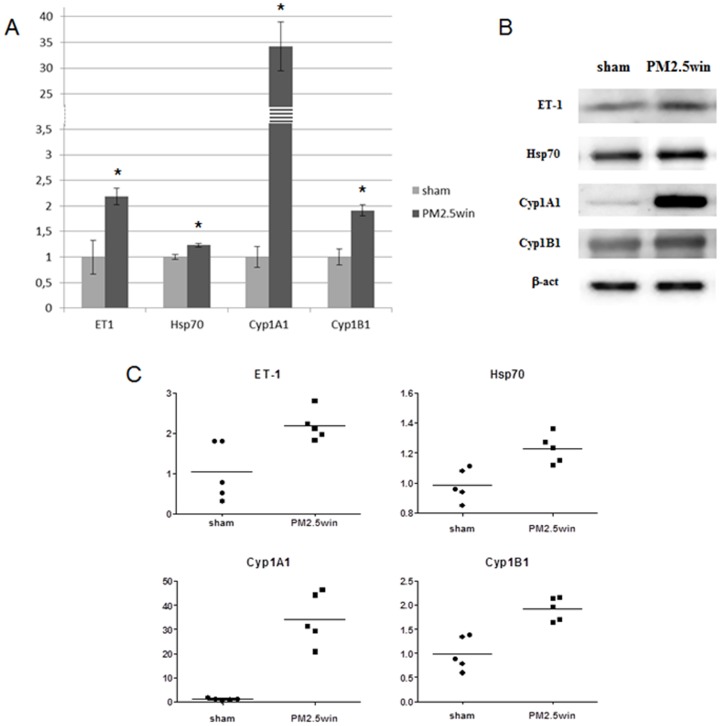
Lung protein analyses. (A) and (B): histograms and representative immunoblottings showing mean± standard error of ET-1, Hsp70, Cyp1A1, Cyp1B1 in lung parenchyma of sham (n = 5) and PM2.5win-treated (n = 5) mice. (C): graphs showing variability among individual animals in significant markers analyzed in lung parenchyma.

**Figure 2:**
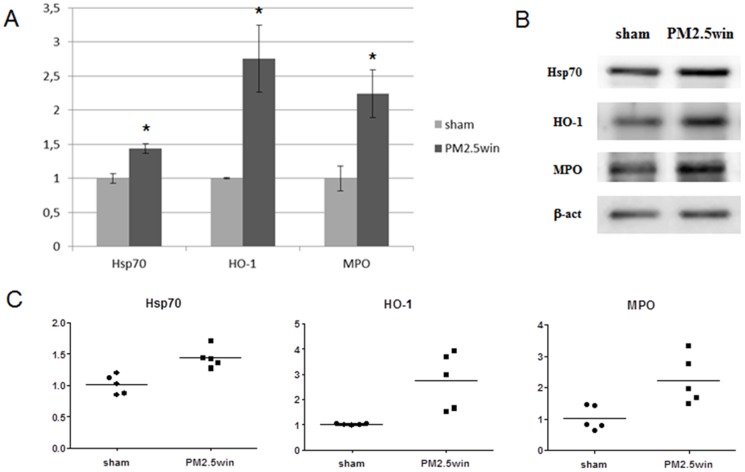
Heart protein analyses. (A) and (B): histograms and representative immunoblottings showing mean± standard error of Hsp70, HO-1, MPO in hearts of sham (n = 5) and PM2.5win-treated (n = 5) mice. (C): graphs showing variability among individual animals in significant markers analyzed in hearts.

In the lung parenchyma of PM2.5win-treated mice, ET-1, Hsp70 and both the cytochromes 1A1 and 1B1 increased comparing to sham. In the heart tissue Hsp-70, HO-1 and MPO increased after PM2.5win treatment. All the other investigated biomarkers both within lungs and heart were not affected by PM2.5win repeated instillations (Tab.3A, Fig.1A, B and C; Tab.3B, Fig.2A, B and C).

**Table 3:** Lung and heart protein analyses.

		Sham (n = 5)		PM2.5win (n = 5)		
		mean	± s.e.	mean	± s.e.	*p*
**A**	ET-1	1.00	0.33	2.18	0.16	*
	Hsp70	1.00	0.05	1.23	0.04	*
	Cyp1A1	1.00	0.2	34.21	4.78	*
	Cyp1B1	1.00	0.16	1.91	0.11	*
	OGG1/2	1.00	0.16	1.47	0.12	ns
	HO-1	1.00	0.09	1.75	0.24	ns
	MPO	1.00	0.25	1.01	0.13	ns
	Casp8-p18	1.00	0.12	1.33	0.06	ns
	Casp3-p17	1.00	0.15	1.23	0.04	ns
	pH3/H3	1.00	0.01	1.29	0.1	ns
**B**	Hsp70	1.00	0.07	1.44	0.07	*
	HO-1	1.00	0.01	2.75	0.49	*
	MPO	1.00	0.18	2.23	0.35	*
	ET-1	1.00	0.47	1.97	0.16	ns
	Cyp1A1	1.00	0.38	0.61	0.06	ns
	Cyp1B1	1.00	0.07	0.84	0.12	ns
	OGG1/2	1.00	0.06	1.18	0.37	ns
	Casp8-p18	1.00	0.15	0.99	0.13	ns
	Casp3-p17	1.00	0.10	1.11	0.06	ns
	pH3/H3	1.00	0.17	0.89	0.04	ns

Table summarizing results in protein markers analyses in lung (A) and heart (B) in sham (n = 5) and PM2.5win-treated mice (n = 5), 24 h after the last intratracheal instillation; the data were normalized for the corresponding β-actin signal in each lane and expressed in relative to sham value. The data are expressed as mean ± s.e. Statistical differences were tested accordingly by non-parametric U Mann-Whitney test. Sham vs. PM2.5-treated: * *p* value <0.05; ns =  not significant.

### Lung histology

**Figure 3:**
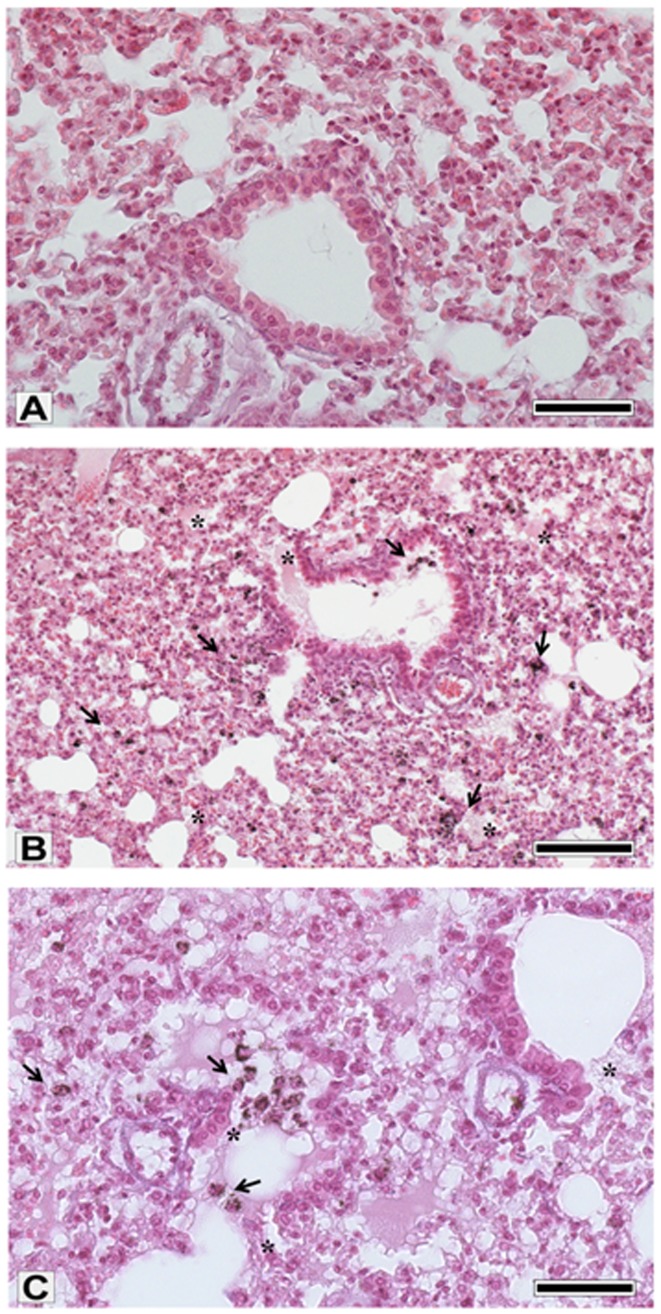
Lung histological analyses. (A): sham lung parenchyma. (B, C): PM2.5win exposed lung parenchyma showing the abundant particulate matter engulfed in phagocytic cells along the lung parenchyma end in cells free in the bronchiolar lumen (arrows), as well as the tissue lesions and exudates (asterisks). A, C, bars  = 50 µm; B, bar  = 150 µm.

Abundant particulate matter was observed engulfed in phagocytic cells along the lung parenchyma and especially within alveolar macrophages (Fig.3). The most evident morphological changes have been found at terminal bronchioles and at adjacent alveolar sacs: here the bronchiolar epithelium often appeared eroded and the surrounding connective tissue was sometimes infiltrated by inflammatory cells. Exudate was evident within the alveolar and bronchiolar spaces, and the alveolar walls often resulted swollen, confirming that PM2.5win affected the air-blood barrier integrity.

### Gene expression profiling of lung and heart RNA samples

#### Global gene expression profiling of lung and heart RNA samples

**Figure 4:**
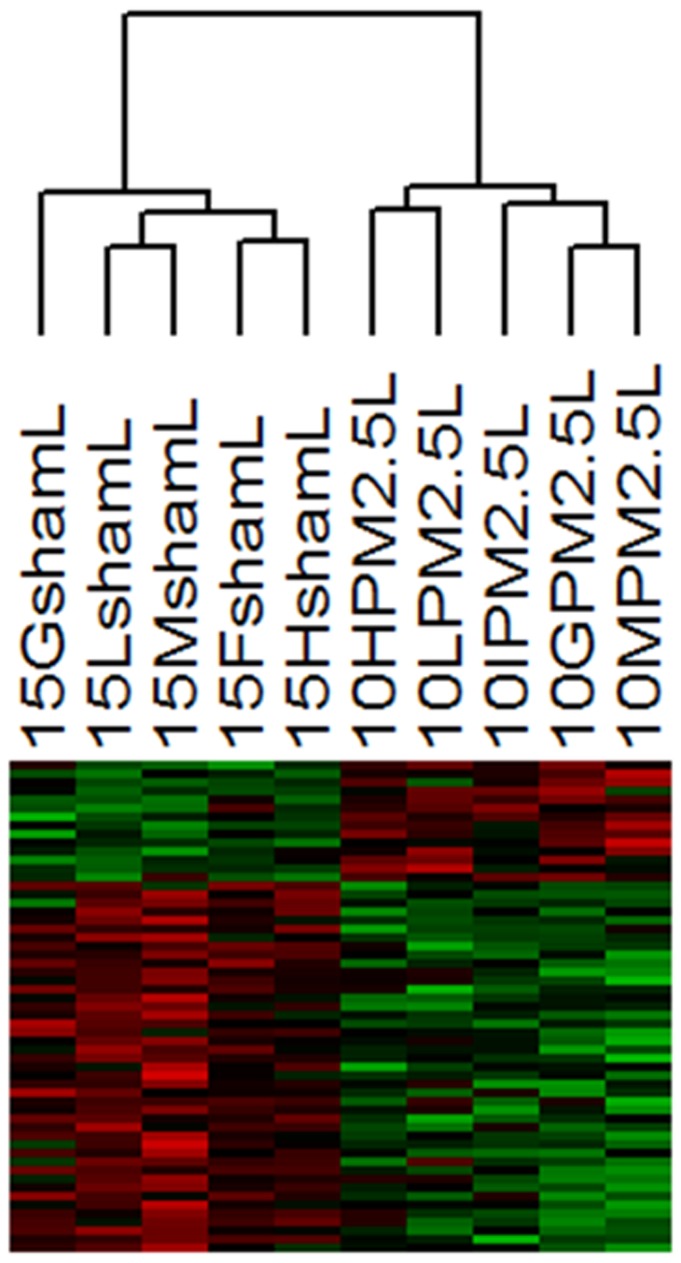
Hierarchical cluster analyses of differentially expressed genes in lung. Hierarchical cluster analyses of 57 DEG between PM2.5win-treated lung (n = 5) and sham (n = 5) mice using dChip software. Each column represents a mouse and each row represents a gene. Red color indicates genes that were up-regulated and green color indicates genes that were down-regulated.

In lungs of PM2.5win-treated mice we found a total of 57 differentially expressed genes (DEG): by means of hierarchical clustering analyses based on DEG (Fig.4 and Fig. S1), we identified 14 up-regulated and 43 down-regulated genes.

**Figure 5:**
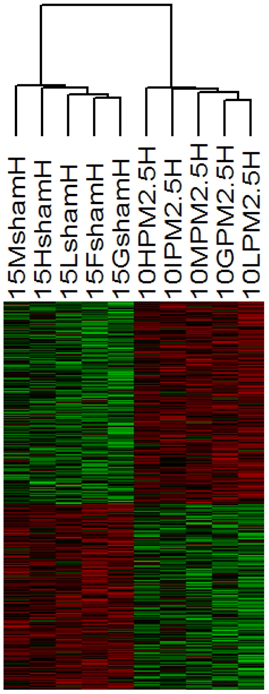
Hierarchical cluster analyses of differentially expressed genes in heart. Hierarchical cluster analyses of 359 DEG between PM2.5win-treated heart (n = 5) and sham (n = 5) mice using dChip software. Each column represents a mouse and each row represents a gene. Red color indicates genes that were up-regulated and green color indicates genes that were down-regulated.

Within lungs, the 90% of the genes displayed 2 to 3 fold-change (Fig.4, Fig. S1 and Table S1). Within heart tissues of mice exposed to PM2.5win, we found a modulation of gene expression of 359 DEG: the hierarchical clustering analyses based on DEG, showing correct discrimination of treated and sham mice (Fig.5 and Fig. S2), identified 181 up-regulated and 178 down-regulated genes. Within hearts, the 89% of DEG displayed a differential modulation of gene expression of 2 to 3 fold-change (Fig.5, Fig. S2 and Table S2).

#### Functional annotation of modulated genes in mouse lung tissues exposed to PM.2.5win

We performed gene ontology enrichments on lung modulated gene list, (Tab.4). Overall, we found a significant GO enrichment for genes involved in cytoskeletal protein and calmodulin binding. Pathways analyses using the list of 57 DEG resulted in 6 KEGG pathways with significant *p* values <0.05 (Tab.5). Among the down-regulated genes, we found five genes (*Ryr2*, *Ryr1*, *Cacna1f*, *Erbb4*, *Pde1c*) involved in calcium signaling. Among the up-regulated genes, we found two members of cytochrome P450 gene family (*Cyp1a1*, *Cyp3a25*) that encoded enzymes involved in an NADPH-dependent electron transport pathway; such enzymes oxidize a variety of structurally unrelated compounds, including steroids, fatty acids, and xenobiotics (Table S1).

**Table 4:** Lung GO enrichments analyses.

Database Category	ID	Description	*p* value	genes #	Total
GO_MF	GO:0008307	structural constituent of muscle	0.000045	7	51
GO_MF	GO:0008092	cytoskeletal protein binding	0.000013	14	784
GO_MF	GO:0005516	calmodulin binding	0.000752	7	208
GO_MF	GO:0003779	actin binding	0.000967	9	415
GO_MF	GO:0005200	structural constituent of cytoskeleton	0.010510	5	127
GO_MF	GO:0005219	ryanodine-sensitive calcium-release channel activity	0.023990	2	5
GO_MF	GO:0090484	drug transporter activity	0.032430	3	32
Pathway	WP383	Striated Muscle Contraction	0.000091	5	38
Pathway	REACTOME_STRIATED_MUSCLE_CONTRACTION	Genes involved in Striated Muscle Contraction	0.001583	4	31
Gene Family	CDH	Cadherins	0.028060	2	33

Functional enrichment of modulated genes in mouse lung exposed to PM.2.5win. Statistical differences were tested accordingly by t-test.

**Table 5:** Lung pathways analsyis.

KEGG ID	p-value	Description	Gene Names
5414	8.81E-07	Dilated cardiomyopathy	*Cacna1f*, *Cacna2d1*, *Dmd*, *Myh6*, *Ryr2*, *Ttn*
5412	1.70E-04	Arrhythmogenic right ventricular cardiomyopathy (ARVC)	*Cacna1f*, *Cacna2d1*, *Dmd*, *Ryr2*
4020	5.01E-04	Calcium signaling pathway	*Cacna1f*, *Erbb4*, *Pde1c*, *Ryr1*, *Ryr2*
2010	1.25E-02	ABC transporters	*Abcb4*, *Abca12*
3320	3.00E-02	PPAR signaling pathway	*Aqp7*, *Fabp4*
5416	3.16E-02	Viral myocarditis	*Dmd*, *Myh6*

Pathways analyses using the list of 57 DEG resulted in 6 KEGG pathways with significant *p* values <0.05. Statistical differences were tested accordingly by t-test.

#### Functional annotation of modulated genes in mouse heart tissues exposed to PM.2.5win

On modulated genes list of heart tissues, we performed a gene ontology enrichments (Tab.6). Overall we found enrichments in several of molecular function GO categories, such as adenyl nucleotide binding, motor activity, hydrolase activity and GTPase binding processes. Moreover we found four modified gene families such as calcium (*Cacna1b*, *Cacna1s*, *Cacna1d*, *Cacna1e*), kinesins (*Kif5a*, *Kif24*, *Kif4a*, *Kif3a*, *Kif20a*, *Kif20b*), mucins (*Muc6*, *Muc5ac*, *Muc2*, *Muc13*), and sodium family (*Scn9a*, *Scnn1g*, *Scn10a*). Pathways analyses using the list of 359 DEG resulted in 15 KEGG pathways with significant *p* values <0.05 (Tab.7). Calcium signaling pathway was the most modulated KEGG term including nine genes: five up-regulated (*Cacna1s*, *Cacna1e*, *Cacna1b*, *Adcy2*, *Gna15*) and four down-regulated (*P2rx3*, *Gnal*, *Nos1*, *Cacna1d*) (Table S2).

**Table 6:** Heart GO enrichments analyses.

Database Category	ID	Description	*p* value	genes #	Total
GO_MF	GO:0017111	nucleoside-triphosphatase activity	0.00002	39	877
GO_MF	GO:0003774	motor activity	0.00007	14	141
GO_MF	GO:0016817	hydrolase activity, acting on acid anhydrides	0.00008	39	926
GO_MF	GO:0005216	ion channel activity	0.00059	23	425
GO_MF	GO:0017016	Ras GTPase binding	0.00220	13	161
GO_MF	GO:0022803	passive transmembrane transporter activity	0.00262	23	464
GO_MF	GO:0015267	channel activity	0.00262	23	464
GO_MF	GO:0000146	microfilament motor activity	0.00383	6	28
GO_MF	GO:0005261	cation channel activity	0.00525	17	288
GO_MF	GO:0031267	small GTPase binding	0.00622	13	177
GO_MF	GO:0051020	GTPase binding	0.01945	13	197
GO_MF	GO:0005245	voltage-gated calcium channel activity	0.02436	6	38
Pathway	P00044	Nicotinic acetylcholine receptor signaling pathway	0.00044	11	89
Gene Family	CACN	Calcium channels	0.00049	4	17
Gene Family	KIF	Kinesins	0.00089	5	39
Gene Family	MUC	Mucins	0.00098	4	20
Gene Family	SCN	Sodium channels	0.01074	3	15

Functional enrichment of modulated genes in mouse heart exposed to PM.2.5win. Statistical differences were tested accordingly by t-test.

**Table 7:** Heart pathways analyses.

KEGG ID	p-value	Description	Gene Names
4020	0.00153	Calcium signaling pathway	*Adcy2*, *Cacna1b*, *Cacna1d*, *Cacna1e*, *Cacna1s*, *Gna15*, *Gnal*, *Nos1*, *P2rx3*
2010	0.00451	ABC transporters	*Abca4*, *Cftr*, *Abca12*, *Abca13*
5146	0.00570	Amoebiasis	*Col11a1*, *Gna15*, *Gnal*, *Lama3*, *Lamc2*, *Muc2*
561	0.00713	Glycerolipid metabolism	*Pnliprp1*, *Lipf*, *Dgki*, *Mboat1*
4512	0.00978	ECM-receptor interaction	*Col11a1*, *Hmmr*, *Itga4*, *Lama3*, *Lamc2*
4530	0.01646	Tight junction	*Myh3*, *Myh4*, *Ppp2r2c*, *Myh13*, *Inadl*, *Myh15*
3450	0.01903	Non-homologous end-joining	*Prkdc*, *Rad50*
5416	0.02469	Viral myocarditis	*Myh3*, *Myh4*, *Myh13*, *Myh15*
4270	0.03305	Vascular smooth muscle contraction	*Adcy2*, *Cacna1d*, *Cacna1s*, *Kcnmb2*, *Kcnu1*
4974	0.03599	Protein digestion and absorption	*Col11a1*, *Col12a1*, *Col17a1*, *Dpp4*
4930	0.03742	Type II diabetes mellitus	*Cacna1b*, *Cacna1d*, *Cacna1e*
300	0.04563	Lysine biosynthesis	*Aass*
4742	0.04577	Taste transduction	*Cacna1b*, *Scnn1g*, *Trpm5*
4710	0.04839	Circadian rhythm - mammal	*Arntl*, *Per1*
5414	0.04985	Dilated cardiomyopathy	*Adcy2*, *Cacna1d*, *Cacna1s*, *Itga4*

Pathways analyses using the list of 359 DEG resulted in 15 KEGG pathways with significant *p* values <0.05. Statistical differences were tested accordingly by t-test.

The most striking aspect of the present study is the twofold to threefold increase in collagen and laminin related genes *Col19a1*, *Col4a3*, *Col12a1*, *Col11a1*, *Col7a1* and *Lama3*; binding to cells via a high affinity receptor, laminin is thought to mediate the attachment, migration and organization of cells into tissues [30] (Table S2). Moreover we found many down regulated motor protein related genes (likely to power actin-based membrane trafficking in many physiologically crucial tissues) within heart of PM2.5win-treated mice. Indeed, *Kif24*, *Dnahc5*, *Kif5a*, *Dnahc8*, *Myo7a*, *Kif4*, *Myh4*, *Myh13*, *Myo5c* expression decreased twofold to threefold (Table S2). Specifically *Myo5c* again plays a role in the regulation of cell morphology and cytoskeletal organization and *Dnah8* is involved in regulation of myosins actin-based motor molecules with ATPase activity while *Myh13* appears to function in the signal transduction from Ras activation to actin cytoskeletal remodeling. *Kif24* and *Myh4* regulates cadherins, calcium dependent cell adhesion proteins which preferentially interact with themselves in a homophilic manner in connecting cells. Interestingly, *Pfkfb1* and *Fpb1* genes, which encodes 6-phosphofructo-2-kinase/fructose-2,6-biphosphatase3 and fructose-1,6-bisphosphatase1, resulted twofold reduced in heart of PM2.5win treated-mice (Table S2).

## Discussion

Air pollution is a major concern for public health, reflecting increased industrialization, energy use, and high road traffic volumes [31]. Numerous adverse health outcomes, in particular cardiovascular and respiratory problems, have been attributed to both long- and short-term air pollution exposure [7], [32]. Several recent works have shown the influence of PM size, composition and/or specific emission sources of particles on biological effects [23], [33], [34], [35], [36] and numerous studies tried to explore the unknown underlying mechanisms of PM-induced adverse health effects [37], [38], [39]. In the current study a mouse model has been used to evaluate the adverse health effects induced by PM2.5win exposure.

### Lungs protein analyses

Lungs are the primary site of exposure to PM. Biochemical analyses performed on BALf and lung parenchyma of PM2.5win-treated mice revealed no significant increase of inflammatory markers, such as differential cells count, cytokines, chemokines and myeloperoxidase, nor of cytotoxic markers, such as LDH or active caspases, comparing to sham. Concerning cells counts, a single PM2.5win intratracheal instillation significantly increased the PMNs percentage 24 h after the treatment [26]; on the contrary, repeated instillations did not change the AMs or PMNs percentage, despite both a not significant increase of PMNs and decrease of AMs. Similarly, after single PM2.5win intratracheal instillation we observed an acute cytotoxic effect [26], while LDH activity resulted unchanged 24 h after the third PM2.5win intratracheal instillation comparing to sham. Actually, we cannot conclude if the acute phase of inflammation is in its reversion phase or if the repeated PM2.5win instillations induce *per se* less inflammation comparing to a single PM2.5win treatment, due to incoming compensatory mechanisms.

Histological evaluation of PM2.5win-exposed lungs fail to disclose massive inflammation: the most significant evidence in PM2.5win-treated lungs was the ubiquitous presence in the alveolar airspace of AMs full of PM2.5win. These data evidenced the active involvement of AMs in PM2.5win clearance. Despite all the above investigated biomarkers of inflammation and cytotoxicity basically resulted unaffected by the PM2.5win repeated instillations, a still ongoing lung dysfunction could be sustained by the here outlined increased levels of Hsp70, Cyp1B1 and ET-1. Indeed lungs showed increased Hsp70 levels consistently with our previous results, concerning a single intratracheal PM2.5win instillation in BALB/c mice. Hsp70 is often associated to urban particulate matter induced ER-stress, as demonstrated by *in-vitro* experiments [40].

The huge amount of PAHs which characterize our PM2.5win samples increased lung cytochrome expression, particularly the Cyp1A1 and Cyp1B1, well-known cytochromes deputized to PAHs metabolism, generating electrophilic metabolites and other reactive oxygen species [41]. In agreement with single instillation treatment [26], however, PAHs metabolism within lungs didn't promote an increase in HO-1 levels. Indeed, despite their lipophilic nature, PAHs are able to enter the bloodstream [42], thus possibly spreading the oxidative stress damage far out from lungs. Finally, ET-1 has been considered able to increase vascular permeability without promoting albumin extravasation in lungs parenchyma [43]. So far, repeated PM2.5win instillations failed to promote significant inflammation or oxidative stress within the alveolar district though sustaining ER-stress as well as endothelial dysfunction. In this situation, we may speculate that the main district involved within lungs of PM2.5win-treated mice could be the alveolar capillary barrier. The endothelial activation may therefore promote an increase of vascular permeability, thus facilitating the translocation of fine particles or chemical compounds from lungs to the bloodstream.

### Heart protein analyses

PM2.5 generally has been associated with an increased risks of myocardial infarction, stroke, arrhythmia, and heart failure exacerbation within hours to days of exposure in susceptible individuals [7].

Consistently with the hypothesis of a most striking effect of fine particles on cardiovascular system, within the heart of our PM2.5win-treated mice MPO, HO-1 and Hsp70 increased comparing to sham. MPO catalyzes the conversion of hydrogen peroxide to hypoclorous acid, which react with NO creating peroxinitrite, with detrimental effects on cell function and thus increasing oxidative stress [44]. Surprisingly, MPO activity may be implicated in the activation of PAHs, such as Benzo[a]Pyrene (BaP), to highly reactive intermediates by ROS generation [45]. As PAHs are largely present within PM2.5win, PAHs not metabolized in the lungs could translocate to the bloodstream being metabolized by MPO thus generating oxidative stress within the heart.

HO-1 role is to catabolize the heme group, generating CO, biliverdin (converted to bilirubin) and Fe^2+^, thus playing a protective role against inflammation and oxidative stress [46] potentially induced by MPO in the heart of our PM2.5win-treated mice. Furthermore, a post-translational down-regulation of cytochromes following the HO-1 induction has been hypothesized, possibly related to a decrease in the heme group bioavailability [47]. This mechanism justify the unchanged cytochromes levels in cardiac parenchyma, even though the presence of PAHs in PM2.5win.

Oxidative stress and ER-stress promote the expression of Hsp70, a well-known protein against inflammation and protein misfolding [48]. Graff et al. [49] demonstrated that the treatment of rat ventricular myocytes with Zn and V induced significant increase in the expression of Hsp70; Zn and V are components of our PM2.5win (Tab.1), and they both might spread in the bloodstream and reach the heart [49], [50], thus explaining the increased Hsp70 in the heart parenchyma of our PM2.5win-treated mice. Moreover, MPO from the bloodstream can be taken up by endothelial cells and once in cardiac tissue it could propagate matrix deposition and adverse ventricular remodeling [51]. Thus, MPO may evolve as an early marker of heart failure that does not simply reflect ventricular dysfunction, but points to humoral and structural alterations that predispose to heart failure [52].

### Lungs and heart gene expression

We then evaluated how approaches at the genomic level would potentially improve our understanding of the air pollutant induced adverse health effects; the knowledge of PM-induced toxic reactions could be useful in order to design strategies better preventing and treating lungs and vascular diseases. Moreover, this gene expression profiling study confirmed the health adverse effects induced by particulate matter exposure both on lung and cardiac tissues.

Among the down-regulated genes in lung tissue, we found five genes *Ryr2*, *Ryr1*, *Cacna1f*, *Erbb4*, *Pde1c* involved in calcium signaling. In particular, it is known that Ryanodine receptors represent a key Ca^2+^ regulatory channel expressed within the microsomal membrane of a wide variety of cells where many xenobiotic molecules are metabolized to bioactive intermediates by the cytochrome P450 system [53]. Intracellular Ca^2+^ has been supposed a key factor in the regulation of Cyp1a1 by various compounds [54]. On the other hand, ErbB4 signaling is important in maintaining adult lung alveolar epithelial cell surfactant synthesis [55].

Moreover, we found that *Cyp1a1* gene was significantly induced in lung mice exposed to PM2.5win versus sham mice. In lungs of PM2.5win-treated mice, the expression of *Cyp1b1* gene was higher than sham mice, but the differences between the two groups did not reach the statistical significance. Moreover we performed a quantitative PCR analyses of *Cyp1b1* gene in lung tissues and we confirmed the up regulation of this gene in PM2.5 treated lung tissue compared to sham (data not shown). PAHs and PM are co-pollutants emitted as by-products of combustion processes and convincing evidence exists for PAHs as a primary toxic component of PM2.5win. As benzo[a]pyrene (BaP) is a potent ligand of aryl hydrocarbon receptors (AhR) [56], we might speculate that PAHs adsorbed on PM2.5win can bind to AhR inducing its translocation to the nuclei and thus resulting in the transactivation of genes of several drug-metabolizing enzymes, such as *Cyp1a1* and *Cyp1b1* [57], [58]. Cyp1A1 and Cyp1B1 are involved in the conversion of BaP itself into an ultimate metabolite, which forms DNA adducts [59], [60]: it has been demonstrated that incubation with BaP increased BaP-DNA adduct levels in rat lung slices [61]. AhR activation and *Cyp1a1*-*Cyp1b1* induction are therefore important indicators of susceptibility to BaP and many studies on lungs tissue demonstrated that *AhR* and *Cyp1a1* are mainly expressed in bronchiolar epithelial cells of the peripheral lung [62], [63], thus indicating that lungs are target site of PAHs toxic effects.

Indeed Longhin et al. [64] provided evidence that Milano PM2.5win induced marked cell cycle alteration, represented by a transient arrest in G2, in bronchial epithelial cells even after 3 h of PM exposure, while DNA adducts have been detected after 24 h. The authors linked this effect to the metabolic activation of PM2.5win organic chemicals, which cause damages to DNA and spindle apparatus; such events could be central to explain the increased lung cancer incidence associated with PM2.5win and deserve further investigations [64]. Lastly, we found a fivefold decrease in *Nppa* gene encoding for Atrial Natriuretic Peptide (ANP). ANP could be synthesized in type II alveolar cells, but while the release by smooth muscle cells in blood has been demonstrated, the role of ANP synthesis in the lungs remains to be determined [65]. Recently, Tankersley et al. [66] proposed that air pollutant could interact with *Nppa* gene and that ANP secretion by lungs could in part contribute to the circulating pool. Thus, the observed down-regulation of NPPA could be crucial in cardiac changes induced by air pollution.

Expression of a number of genes has been investigated for their potential prognostic value in human heart failure [67], [68]. It has been evidenced that the onset of heart failure triggers a mechanism that up-regulates fibronectin and collagen gene expression [69]. Since increases in fibrillar collagen in the heart interstitium contribute to tissue stiffness, increases in fibronectin and collagen gene expression may contribute to heart impaired function. Indeed, in mice exposed to PM2.5win, the most striking aspect is the twofold to threefold increase in collagen and laminin related genes (*Col19a1*, *Col4a3*, *Col12a1*, *Col11a1*, *Col7a1* and *Lama3*). These results are consistent with the concept that some myocytes in hearts of PM2.5 exposed mice are putative prone to exhibit a nascent hypertrophic response [67]. Moreover, we found many down regulated motor protein related genes within the heart of PM2.5win-treated mice and this surprisingly well correlates with the findings obtained from the SHR model of heart failure [70].

In ventricular myocytes, a multitude of channels are involved in the intracellular Ca^2+^ regulation mechanism [71]. We found that calcium signaling pathway was the most modulated, as nine genes resulted in KEGG pathways analyses: five up-regulated (*Cacna1s*, *Cacna1e*, *Cacna1b*, *Adcy2*, *Gna15*) and four down-regulated (*P2rx3*, *Gnal*, *Nos1*, *Cacna1d*). Dysregulation of ion channel gene expression in heart tissues potentially contributes to altered myocardial handling of Na^+^ and Ca^2+^ and subsequent Ca^2+^ overload, tissue hyperexcitability, and arrhythmogenesis. Indeed, cardiac function depends on the appropriate timing of contraction and the appropriate beating rate in each region. Excitation–contraction (EC) coupling comprises processes involved in the Ca^2+^ activation of contractile proteins and the subsequent removal of Ca^2+^ facilitating relaxation; therefore, alterations of EC-coupling may play a critical role in the pathophysiology of myocardial failure [72], [73], [74].

## Conclusions

Air pollution remains an important public health worldwide problem. There is now a strong imperative to use the best air pollution *in-vitro* and *in-vivo* models, combined with genomics, to identify the key pathways involved in mechanisms of health adverse effects induced by air pollution. The current study extends our previous findings, showing that repeated instillations of fine particulate matter trigger systemic adverse effect. The study of genomic responses will improve understanding of disease mechanisms and enable future clinical testing of interventions against the toxic effects of air pollutants. At present-day levels, PM2.5win likely poses an acute threat principally to susceptible people, even if seemingly healthy, such as the elderly and those with unrecognized coronary artery or structural heart disease. While there is clearly an important public health initiative to contain rising levels of air pollution, it is also important to develop strategies minimizing the damaging effects of air pollutant exposure.

## Supporting Information

Figure S1 **Lung differentially expressed gene (DEG) distribution.** Distribution of 57 DEG according the *p* value (horizontal axes) and Fold Change value (vertical axes).
(TIF)

Figure S2 **Heart differentially expressed gene (DEG) distribution.** Distribution of 359 DEG according the *p* value (horizontal axes) and Fold Change value (vertical axes).
(TIF)

Table S1 **Global gene expression in lung tissue.** List of differentially expressed gene (DEG) of lung exposed to PM.2.5win; in lung tissue of PM2.5win-treated mice 14 up- and 43 down-regulated genes have been found.
(XLS)

Table S2 **Global gene expression in heart tissue.** List of differentially expressed gene (DEG) of heart exposed to PM.2.5win; in heart tissue of PM2.5win-treated mice 181 up- and 178 down-regulated genes have been found.
(XLS)
