# Supplementary figures and images for: Health Risk Assessment for Air Pollutants: Alterations in Lung and Cardiac Gene Expression in Mice Exposed to Milano Winter Fine Particulate Matter (PM2.5)
Source: PLoS One. 2014 Oct 8;9(10):e109685. doi: 10.1371/journal.pone.0109685 (PMC4190364; doi:10.1371/journal.pone.0109685)

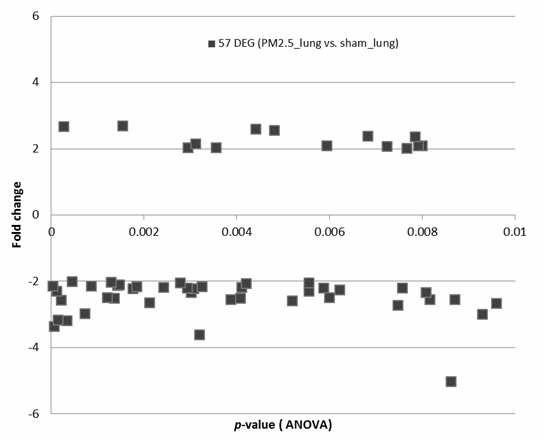

Supplement: Figure S1 — Lung differentially expressed gene (DEG) distribution. Distribution of 57 DEG according the p value (horizontal axes) and Fold Change value (vertical axes). (TIF) [file pone.0109685.s001.tif]

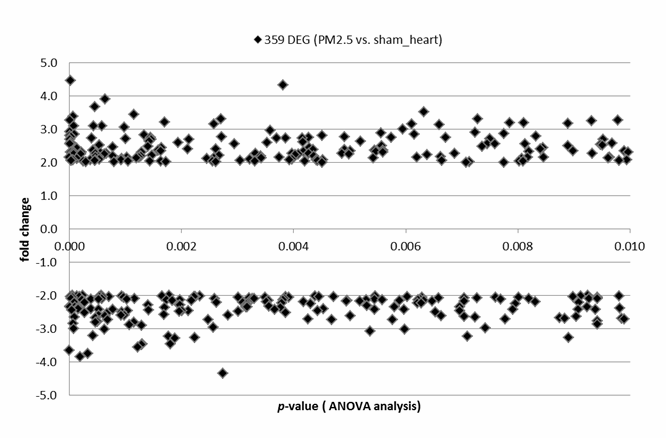

Supplement: Figure S2 — Heart differentially expressed gene (DEG) distribution. Distribution of 359 DEG according the p value (horizontal axes) and Fold Change value (vertical axes). (TIF) [file pone.0109685.s002.tif]
